# Immune checkpoint inhibition: prospects for prevention and therapy of hepatocellular carcinoma

**DOI:** 10.1038/cti.2017.47

**Published:** 2017-11-10

**Authors:** Caryn L Elsegood, Janina EE Tirnitz-Parker, John K Olynyk, George CT Yeoh

**Affiliations:** 1 School of Biomedical Science, Curtin Health Innovation Research Institute, Curtin University, Perth, Western Australia, Australia; 2 Department of Gastroenterology and Hepatology, Fiona Stanley and Fremantle Hospitals, South Metropolitan Health Service, Murdoch, Western Australia, Australia; 3 School of Health and Medical Sciences, Edith Cowan University, Joondalup, Western Australia, Australia; 4 School of Chemistry and Biochemistry, The University of Western Australia, Crawley, Western Australia, Australia; 5 Harry Perkins Institute of Medical Research, QEII Medical Centre, Nedlands and Centre for Medical Research, The University of Western Australia, Crawley, Western Australia, Australia

## Abstract

The global prevalence of liver cancer is rapidly rising, mostly as a result of the amplified incidence rates of viral hepatitis, alcohol abuse and obesity in recent decades. Treatment options for liver cancer are remarkably limited with sorafenib being the gold standard for advanced, unresectable hepatocellular carcinoma but offering extremely limited improvement of survival time. The immune system is now recognised as a key regulator of cancer development through its ability to protect against infection and chronic inflammation, which promote cancer development, and eliminate tumour cells when present. However, the tolerogenic nature of the liver means that the immune response to infection, chronic inflammation and tumour cells within the hepatic environment is usually ineffective. Here we review the roles that immune cells and cytokines have in the development of the most common primary liver cancer, hepatocellular carcinoma (HCC). We then examine how the immune system may be subverted throughout the stages of HCC development, particularly with respect to immune inhibitory molecules, also known as immune checkpoints, such as programmed cell death protein-1, programmed cell death 1 ligand 1 and cytotoxic T lymphocyte antigen 4, which have become therapeutic targets. Finally, we assess preclinical and clinical studies where immune checkpoint inhibitors have been used to modify disease during the carcinogenic process. In conclusion, inhibitory molecule-based immunotherapy for HCC is in its infancy and further detailed research in relevant *in vivo* models is required before its full potential can be realised.

## Introduction

Primary liver cancer is the sixth most prevalent cancer globally, but importantly the second most common cause of cancer-related death due to limited treatment options.^1^ The risk of adult primary liver cancer is considerably enhanced by cirrhosis resulting from viral hepatitis (hepatitis B virus (HBV) and hepatitis C virus (HCV)), alcohol, obesity, metabolic liver diseases and aflatoxin exposure. Paediatric primary liver cancer generally results from genetic conditions, such as Beckwith–Wiedemann syndrome, hemihypertrophy and familial adenomatous polyposis, and inborn metabolic errors, such as tyrosinaemia, alpha-1 antitrypsin deficiency and glycogen storage disease type 1. Resection and percutaneous local ablation are the only treatment options for early-stage tumours. Repeated transarterial chemoembolisation is used for intermediate stage, while oral sorafenib is the gold-standard treatment for advanced hepatocellular carcinoma (HCC) with only modest improved survival time.^2^ Thus it is imperative that new alternatives are developed to limit liver cancer development or to treat advanced liver cancer.

HCC, cholangiocarcinoma (or bile duct cancer), primary hepatic angiosarcoma and hepatoblastoma represent the four main subtypes of primary liver cancer. Rare variants are tumours with combined hepatocellular and cholangiocellular features, referred to as a mixed hepatocellular cholangiocarcinoma.^3^ HCC is the most studied subtype and accounts for 85–90% of all primary liver cancers. There is evidence to support its origin from hepatocytes or a liver stem/progenitor cell in both adults and children.^4^ Cholangiocarcinoma is a heterogeneous malignancy that develops in the biliary tree of adults and is classified as intrahepatic, perihilar or distal based on the anatomical location.^5^ Primary hepatic angiosarcoma is an extremely rare soft tissue sarcoma in which pleomorphic endothelial cells grow into vascular spaces, including sinusoids and terminal hepatic venules.^6^ Hepatoblastoma is similarly a very rare paediatric primary liver cancer thought to arise from a hepatocyte precursor known as a hepatoblast, which is present during fetal liver development.^7^

The original six hallmark features of cancer focussed on tumour cell features that enabled survival, proliferation and dissemination.^8^ Importantly, the immune system has now also been recognised to be central to tumorigenesis in an expanded roster of hallmarks of cancer.^8^ Accordingly, a number of strategies to inhibit carcinogenesis are being developed, which target distinct immunological mechanisms.^9^ The immune system can (i) suppress viral-induced tumours by protecting the host against infection,^9^ (ii) prevent establishment of a chronic inflammatory environment that promotes cancer by inducing genetic instability and mutation in target cells^9, 10^ and (iii) eliminate tumour cells that often co-express ligands for activating innate immune cell receptors and tumour antigens that are recognised by lymphocyte receptors.^9^ However, importantly, the tolerogenic nature of the liver presents unique and specific challenges to suppressing hepatic tumour development.

Oncolytic immunotherapy has been explored in many types of tumours. Immunotherapy for HCC, though, is relatively underexplored. Interleukin-12 (IL-12) cytokine administration and IL-12-based gene and cell-based therapies have been used to treat HCC in preclinical studies.^11, 12, 13^ Granulocyte macrophage colony-stimulating factor-based gene therapy has been used to successfully reduce tumour burden in HCC patients.^14^ However, it is unclear if the efficacy was due to immune- or viral-based oncolysis. Adoptive transfer of chimeric antigen receptor-modified T cells is presently being examined as a possible therapeutic for HCC.^15^ There is similarly much excitement with the advent of monoclonal antibody-based therapy to block immune-inhibitory molecules, such as programmed cell death protein-1 (PD-1), programmed cell death 1 ligand 1 (PDL-1) and cytotoxic T lymphocyte antigen 4 (CTLA4), which prevent T cells from killing tumour cells. This therapy is now widely used in the clinic to treat solid tumour melanoma^16^ and is in the early stages of evaluation in the setting of HCC. Importantly, the immune response to blocking of inhibitory molecules can be sustained beyond the prescribed treatment duration.

In this review, we will explore how the immune system regulates the development of the most common liver cancer, HCC, with a focus on the inhibitory receptors known as immune checkpoint inhibitors. We will first review the T-cell subsets present in the liver together with basic concepts of T-cell activation, differentiation and exhaustion. We will briefly discuss mechanisms that contribute to hepatic immune tolerance as well as cytokines that regulate the tolerance. Immune inhibitory molecules have primarily been viewed as agents that inhibit immune responses to tumour cells. However, it is now apparent that their expression is also upregulated during viral infection and chronic inflammation. Thus we will focus on how viral hepatitis, chronic inflammation and HCC use the inhibitory molecules to subvert the immune system’s ability to prevent hepatic carcinogenesis. Finally, we will explore how the new inhibitory molecule-based immunotherapies may be utilised to prevent HCC development or treat advanced HCC.

## Immune cells and cytokines involved in HCC development

The immune system has a central role in the progression of chronic liver disease and HCC development with the relative contributions and responses of regulatory, helper and cytotoxic effector T cells central to determining whether chronic liver disease progresses to HCC. In particular, cytotoxic T cells are essential for targeting and destroying infected or tumorigenic hepatic cells while regulatory T cells dampen cytotoxic T-cell responses. The T-cell subsets present are determined by contextual information such as the cytokine milieu and strength of antigen stimulation. Below, we examine the key basic concepts of T-cell biology and cytokine regulation of hepatic tolerance that are important for HCC development.

### T-cell activation and differentiation

Naive T cells activate and clonally expand in response to antigen-presenting cell (APC)-expressed major histocompatibility complex (MHC)-I or MHC-II binding to the T-cell receptor (TCR) of CD8^+^ or CD4^+^ T cells, respectively. In general, the clonally expanded CD4^+^ T cells differentiate into regulatory and helper T-cell subsets in response to the cytokines they are exposed to and as defined by their transcription factor expression and cytokine secretion. The most common hepatic CD4^+^ T-cell subsets are T helper type 1 (T_h_1), T_h_2, T_h_9 and T_h_17 helper T cell and regulatory T cells (T_regs_) subsets. CD8^+^ T cells differentiate into cytotoxic T cells and subsequently into memory T cells. The cytokine combinations that induce each subset *in vitro*, together with the master transcription factor expressed, characteristic cytokines secreted and global function(s) of each T-cell subset, are described below.

#### T_h_1 cells

IL-12 together with interferon (IFN)-γ induce naive CD4^+^ T cells to differentiate into T_h_1 cells, which express the T-bet transcription factor and secrete IFN-γ and tumour necrosis factor (TNF).^17, 18^ T_h_1 cells promote cell-mediated immunity and activate clearance of tumour cells and intracellular pathogens as a result of production of the pro-inflammatory cytokines.^17^

#### T_h_2 cells

T_h_2 cells are produced from naive CD4^+^ T cells in response to IL-4 and IL-33, express GATA-3 transcription factor and secrete IL-4, IL-5, IL-10, IL-13 and IL-25.^17, 18^ T_h_2 cells suppress T_h_1-driven inflammation and associated tissue damage and remove extracellular parasites such as helminths and *Schistosoma* as a result of recruitment of eosinophils, mast cells and basophils.^17^

#### T_h_9 cells

T_h_9 cells are produced from naive CD4^+^ T cells by stimulation with transforming growth factor (TGF)-β together with IL-4, express PU.1 transcription factor and secrete IL-9 and IL-10.^18^ T_h_9 cells promote T_h_2 responses in murine atopic diseases, are pro-inflammatory in ulcerative colitis and antitumoral in solid tumour models.^18^ Peripheral blood T_h_9 cell numbers are elevated in cirrhotic patients and are positively associated with the severity of cirrhosis.^19^ However, the nature of the T_h_9 cells’ role in chronic liver disease remains undetermined.

#### T_h_17 cells

T_h_17 cells are produced from naive CD4^+^ T cells in the presence of TGF-β together with IL-6 and IL-21 in mice or IL-23 in humans.^18^ T_h_17 cells express retinoic acid–related orphan receptor γt transcription factor and produce IL-17, IL-21, IL-22 and TNF. IL-17 promotes endothelial cell expression of granulocyte macrophage colony-stimulating factor (or colony-stimulating factor-2) and/or granulocyte colony stimulating factor (or colony-stimulating factor-3) and thus inflammatory neutrophil and monocyte recruitment.^20^ T_h_17 cells may have a critical role in the development of liver diseases such as viral hepatitis and alcoholic liver disease as well as HCC where their numbers correlate with angiogenesis and poor prognosis.^21, 22^ Indeed, IL-17 mediates the spontaneous HCC that develops by 65 weeks of age in mice expressing human unconventional prefoldin RPB5 interactor (URI) in hepatocytes.^23^ However, similar to T_h_9 cells, T_h_17 cells’ role is yet to be determined. T_h_17 cells are also important for clearance of extracellular bacteria and fungi.^17^

#### Regulatory T cells

IL-2 in concert with TGF-β induces naive CD4^+^ T-cell differentiation to T_regs_ *in vitro*.^17^ T_regs_ express the Foxp3 transcription factor and secrete IL-10 and TGF-β, which both suppress the inflammatory functions of monocytes, macrophages, dendritic cells (DCs) and B cells.^24^ T_regs_ also promote further differentiation of naive CD4^+^ T cells to T_regs_ as a result of TGF-β production. Importantly, T_regs_ maintain homeostasis and immune tolerance.^25^ However, an overabundance of T_regs_ promotes an immunosuppressive environment.^24^

T_regs_ may be derived *in vivo* from naive CD4^+^ T cells either in the thymus (thymus-derived T_regs_) or in peripheral tissues, such as lymph nodes, spleen and liver (peripheral-derived T_regs_). The definitive origin of T_regs_ in the peripheral tissues including liver can, however, not be ascertained owing to the current lack of defining markers.^26^ Thus, in this review, we will refer to them collectively as T_regs_.

#### Cytotoxic and memory CD8^+^ T cells

Naive CD8^+^ T cells clonally expand in response to IL-12, or type I IFNs (IFN-α, IFN-β).^27^ Cytotoxic T-cell numbers then diminish during clearance of the infection, and the remaining cells mature into memory CD8^+^ T cells. IL-7 and IL-15 maintain memory T-cell numbers in the absence of antigen.^28^ Cytotoxic antigen-specific CD8^+^ T cells express TNF, IFN-γ, Fas ligand, granzyme B and perforin and kill virus-harbouring or cancer cells expressing a specific antigen. *In vivo*, optimal CD8^+^ T-cell function usually requires help from CD4^+^ T cells, including T_h_1 cells, in the form of regulatory cytokines (for example, IL-21) and chemokines as well as activating DCs via CD40:CD40 ligand interactions.^27, 29^ Thus the absence of CD4^+^ T cells results in defective CD8^+^ T-cell production, reduced CD8^+^ memory cells and the inability to mount a vigorous secondary immunogenic response.^30^

### Regulation of T-cell activation strength

**Figure 1:**
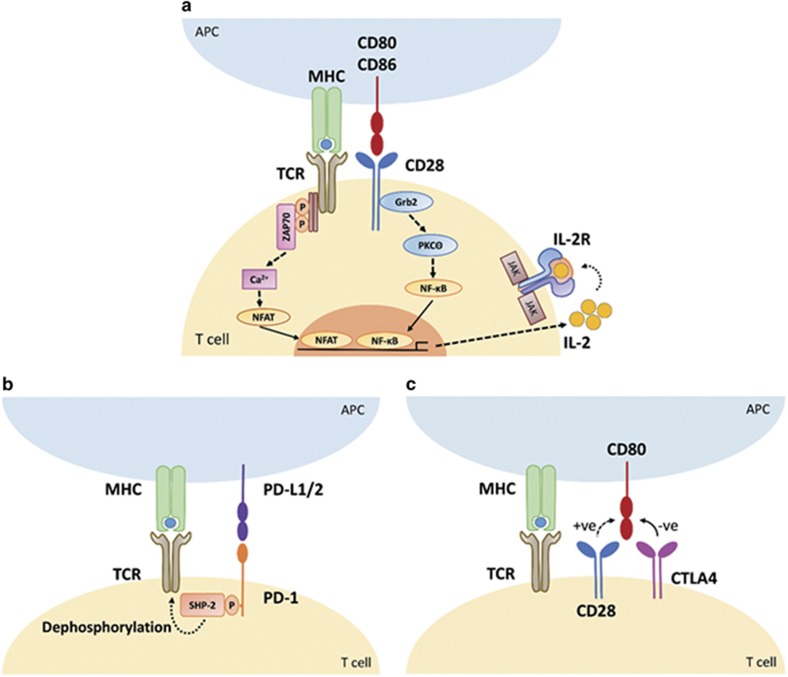
(**a**) T-cell activation is promoted by antigen presented to the TCR by APC-expressed MHC. The strength of T-cell activation is enhanced by APC-expressed CD80 or CD86 binding to T cell-expressed CD28. TCR and CD28 downstream signalling promote nuclear factor (NF)-activated T cells (NFAT) and NF-κB nuclear relocation, respectively, which synergise to promote IL-2 production and T-cell survival by IL-2 autocrine signalling to the IL-2R. (**b**) T-cell activation strength is diminished by APC-expressed PD-L1 or PD-L2 binding to T-cell-expressed PD-1 to promote dephosphorylation of the TCR and reduce TCR downstream signalling and IL-2 production. (**c**) T-cell-expressed CTLA4 binds to APC-expressed CD80 with higher affinity than CD28 and attenuates CD28 downstream signalling and IL-2 production. JAK, Janus-activated kinase.

The strength of T-cell activation is controlled by the amount of antigen presented to the TCR and by positive and negative co-receptors. The most commonly described APC-expressed co-stimulatory receptors are CD80 (or B7-1) and CD86 (or B7-2), which engage T cell-expressed CD28 to induce T-cell activation, as a result of T-cell survival promoted by autocrine IL-2 signalling (Figure 1a).^31^ The functions of other co-stimulatory receptors have recently been reviewed extensively by Chen and Flies.^31^

Activation of the TCR is diminished by APC-expressed co-inhibitory receptors such as PD-L1 (or B7-H1 or CD274) and PD-L2 (or B7-DC or CD273), which both interact with T cell-expressed PD-1 (or CD279) and dephosphorylate the TCR (Figure 1b). Further, T-cell activation can be downregulated by APC-expressed CD80 binding to CTLA4 or CD152, which outcompetes costimulatory CD28 for binding to APC-expressed CD80 as a consequence of higher binding affinity (Figure 1c). APC-expressed PD-L1 binding to T-cell-expressed CD80 and APC-expressed CD80 binding to T-cell-expressed PD-L1 also attenuates T-cell activation although the precise mechanism is unknown.^32^ Further, APC-expressed galectin 9 and MHC-I/MHC-II can bind to T-cell-expressed T-cell immunoglobulin and mucin-domain containing-3 (Tim-3) and lymphocyte-activation gene 3 (Lag-3/CD223), respectively, to attenuate T-cell activation. Other inhibitory receptors have also recently been reviewed by Chen and Flies.^31^

### T-cell exhaustion

T-cell exhaustion has been described for both CD4^+^ and CD8^+^ T-cell populations and occurs in response to persistent antigen exposure and/or inflammation. Exhausted T cells have sustained expression of co-inhibitory receptors, reduced effector function and an altered transcriptional state compared with functional effector or memory T cells.^33^ Importantly, exhausted T cells cannot mature into memory T cells. T-cell exhaustion is characteristic of chronic viral infection and tumorigenesis.

#### Induction of T-cell exhaustion

A key feature of T-cell exhaustion is the chronic exposure of the TCR to antigen, with the extent of exhaustion correlating with the level and duration of antigen exposure. Inhibitory receptor signalling through PD-1 also downregulates T-cell activation.^34^ Another contributing factor to CD8^+^ T-cell exhaustion may be CD4^+^ T_helper_ cell exhaustion with concomitant enhanced IL-10 and IL-21 secretion.^35^

T-cell exhaustion is a stepwise process where the ability of the T cells to produce IL-2 and proliferate is affected first, followed by TNF secretion in the intermediate stages, with IFN-γ secretion most resistant to inactivation.^33^ At the same time, there is a progressive increase in the number and expression levels of co-inhibitory receptors such as PD-1, CTLA4 and LAG3, over and above the levels induced during differentiation, in addition to altered transcription factor expression. In the final phase, the antigen-specific T cell can be deleted. Importantly, it is now widely appreciated that exhausted T cells are distinct from memory T cells and require antigen for survival. Exhausted T cells are refractory to IL-7 and IL-15, which promote memory T-cell survival.^33^ For further extensive review of the T-cell exhaustion process, please see Wherry and Kurachi.^36^

#### Reversal of T-cell exhaustion

T-cell exhaustion can be reversed by antibody neutralisation of the co-inhibitory receptors known universally as immune checkpoint inhibitors and that reduce T-cell activation. Neutralising antibody blockade of PD-1 or PD-L1 in the chronic murine lymphocytic choriomeningitis virus (LCMV) infection model established that it was possible to reverse T-cell exhaustion.^37^ Specifically, PD-1 blockade increased proliferation, cytokine production and cytolytic activity of hepatic and splenic CD8^+^ cytotoxic T cells in chronic murine LCMV infection resulting in reduced viral titre. Increased efficacy of treatment can be gained by combining blockade of PD-L1 and other inhibitory molecules, such as Lag-3 and Tim-3.^38, 39^

Blockade of inhibitory molecules has also been combined with therapies directed at extrinsic pathways, including IL-2 or IL-10. IL-2 administration synergises with PD-L1 blockade to enhance virus-specific CD8^+^ T-cell responses and reduce viral titre in the chronic LCMV infection mode despite a concomitant increase in T_reg_ numbers.^40^ IL-10 can upregulate PD-L1 expression, and blockade of both IL-10 signalling and PD-L1 can also synergistically enhance cytotoxic CD8^+^ T-cell expression of IFN-γ and TNF and likewise reduce LCMV viral titre.^41^ Additionally, T_reg_-depletion synergises with PD-1 or Tim-3 blockade to augment CD8^+^ T-cell responses in the murine Friend Virus model.^42, 43^ These first successful attempts at combined therapies in preclinical hepatic viral models highlighted their potential clinical value in a patient setting.

### Hepatic tolerance and T-cell regulation

The liver is the only non-lymphoid tissue in which naive CD4^+^ and CD8^+^ T cells produced in the thymus can be activated independently of the spleen and lymph nodes.^44, 45^ Naive CD4^+^ and CD8^+^ T cells are activated in the liver by non-conventional APCs in addition to DCs.^46^ Non-conventional hepatic APCs expressing MHC-I and MHC-II include Kupffer cells (KCs or resident macrophages), liver sinusoidal endothelial cells (LSECs) and hepatic stellate cells.^47^ Hepatocytes can also cross-present antigen but only to CD8^+^ T cells as they express MHC-I and not MHC-II.^47^ Hepatic stellate cells, though, may present the antigen via transfer to LSECs in a process akin to trogocytosis, the process of intercellular plasma membrane protein exchange.^48^

Under homeostatic conditions, the liver is continuously exposed to harmless blood-borne non-self antigens originating from dietary nutrients, intestinal commensals and cellular debris. Thus specialised mechanisms must be used to maintain hepatic tolerance. Conventional and non-conventional APCs in the liver typically express low levels of MHC molecules as well as CD80 and CD86 and are accordingly weak T-cell activators.^49^ KCs are key regulators of tolerance through their ability to produce heightened amounts of IL-10 in response to lipopolysaccharide that then downregulates expression of MHC molecules, CD80 and CD86 by the other APCs.^50, 51^ Additionally, hepatic DCs differ from other tissue DCs in that they also produce elevated amounts of IL-10.^52^ Naive CD8^+^ T cells also promote PD-L1 expression by LSECs, which in turn suppresses IL-2 production by CD8^+^ T cells.^53^ LSECs indirectly promote tolerance by reducing DC expression of co-stimulatory molecules and IL-12 in a process known as ‘vetoeing’.^54^ Thus homeostatic hepatic-naive T-cell activation is low, resulting in tolerance.

#### Cytokine regulation of hepatic tolerance

Hepatic tolerance and breaking of tolerance (or hepatic immunity) is also modulated by microenvironment factors produced by surrounding APCs and T cells themselves, including anti- and pro-inflammatory cytokines, such as IL-10, TGF-β, IFN-γ and TNF. IL-10 induces tolerance via effects on T cells and dampening inflammatory responses. TGF-β is a key cytokine in maintenance of immune homeostasis or tolerance, but elevated levels can promote detrimental tolerance or immunosuppression in the context of tumour cell immune surveillance. IFN-γ and TNF are both thought to break immune tolerance, although chronic exposure to TNF may induce immunosuppression. The effects of these cytokines on hepatic tolerance are briefly discussed below.

##### Interleukin-10

Hepatic IL-10, produced by KCs, DCs, LSECs, hepatic stellate cells, T cells^55^ and tumour-induced myeloid-derived suppressor cells directly and indirectly promotes hepatic tolerance.^56^ IL-10 directly inhibits CD4^+^ T-cell activation and thus reduces effector T-cell numbers^57^ and DC-primed cytotoxic CD8^+^ T-cell function.^29^ Hepatic T-cell activation is also indirectly reduced by IL-10 as a result of downregulation of MHC-II and co-stimulatory CD80/CD86 molecules on KCs and LSECs with a subsequent reduction of their T-cell stimulatory capacity.^58^ Further, IL-10 downregulates nuclear factor-κB activity with concomitant reduction of inflammatory cytokines, including TNF, IL-6, IL-8 and IL-12, and thus alleviates T-cell activation.^59^

###### Transforming growth factor-β

Hepatic TGF-β, produced by KCs, DCs, LSECs, hepatic stellate cells, T_regs_, natural killer (NK) T cells and myeloid-derived suppressor cells also promotes hepatic tolerance.^60^ TGF-β is critical for naive CD4^+^ T-cell differentiation to T_regs_ and inhibits differentiation of naive CD4^+^ T cells to pro-inflammatory T_h_1 and T_h_2 T-cell subsets as a result of inhibiting expression of the T_h_1 and T_h_2 essential transcription factors, T-bet and GATA3, respectively.^61, 62^ Additionally, TGF-β inhibits differentiation of naive CD8^+^ T cells to cytotoxic CD8^+^ T cells independently of Smad3.^63, 64^ Further, TGF-β inhibits cytotoxic CD8^+^ T-cell activity as a result of inhibition of perforin and IFN-γ expression.^65^

####### Interferon-γ

The primary sources of IFN-γ in the liver are NK cells, CD4^+^ T_h_1, cytotoxic CD8^+^ T cells and NKT cells.^66^ IFN-γ secretion by NK and T cells is promoted by IL-12, IL-18 and IFN-γ itself in a feedforward loop, but production in NKT cells is independent of cytokine signalling.

IFN-γ is required for T_h_1 immunity as it induces expression of the T_h_1 master transcription factor, T-bet, resulting in naive CD4^+^ T-cell differentiation to T_h_1 cells. *In vitro*, IFN-γ inhibits differentiation of T_h_2 and T_h_17 subsets. However, paradoxically, T_h_1 and T_h_17 cells are often found in close association in pathology. *In vivo*, T_h_1 cell production of IFN-γ can trigger myeloid APC production of IL-1 and IL-23 that promote T_h_17 differentiation, at least in psoriasis.^67^ IFN-γ also activates innate immune cells such monocytes, macrophages and neutrophils to break tolerance and enhance antiviral effects. IFN-γ promotes M1 macrophage polarisation and boosts innate immune cell production of reactive oxygen species by enhancing the expression of nitric oxide synthase 2, nitric oxide synthase 2 cofactors and NADPH oxidase components.^68^ IFN-γ can also promote an immunosuppressive phenotype by synergising with TGF-β to amplify Foxp3 expression by CD4^+^ T cells and enhance T_reg_ cell differentiation.^68^ However, prolonged IFN-γ exposure in IFN-γ-overexpressing mice reduced HCC in DEN-induced HCC model as a result of enhancing hepatocyte apoptosis.^69^

***Tumour necrosis factor***

Hepatic TNF is produced by KCs, monocytes, DCs, B cells and T_h_1 and T_h_17 cells. TNF has pleiotropic roles in immune responses and its effects may depend upon the context, amount of TNF present and the involvement of either or both TNF receptors, which often display disparate and opposing roles.^70^ The effect of hepatic TNF exposure on immune responses is relatively unexplored. Acute TNF signalling via the TNF receptors 1 and 2 results in apoptosis or inflammation, respectively. Chronic TNF exposure in primary sclerosing cholangitis patients, though, promotes immunosuppression with reduced T-cell proliferation and cytotoxic T-cell activity.^71^ The immunosuppression may be due to TNF-induced nuclear factor-κB-mediated upregulation of CD4^+^ T-cell expression of PD-1 as demonstrated in chronic LCMV infection of neonatal mice.^72^

## Subversion of the immune system during HCC development

The immune system can prevent cancer by protecting against viral infection, reducing inflammation and/or eliminating tumour cells as a result of recognition of tumour antigens.^9^ Below we will discuss how hepatic immune surveillance is subverted to promote chronic viral infection and inflammation, both of which can amplify liver cancer development. We also examine how hepatic tumorigenic cells can avoid elimination by the immune system, resulting in HCC progression.

### Viral-induced chronic liver disease

Globally, HBV and HCV are the leading viral risk factors for liver cancer development. As previously discussed, the tolerogenic nature of the liver invariably means that it does not generate a robust immune response to acute HBV and HCV infections. Thus HBV and HCV trigger persistent and chronic inflammatory infections where CD4^+^ helper T-cell function is impaired, numbers of exhausted CD8^+^ cytotoxic T cells with suppressed IL-2, TNF and IFN-γ secretion are reduced and NK cell dysfunction is observed.^73^

The importance of the CD4^+^ T helper cells in HBV and HCV clearance has been highlighted in the chimpanzee model. In a study by Greyer *et al.*,^29^ CD4^+^ T-cell depletion prior to HBV infection or HCV reinfection gave rise to persistent infection due to reduced responses by the integral CD8^+^ cytotoxic T cells.^74^ Naive CD8^+^ T cells are ‘primed’ by CD4^+^ T cell-induced DC production of IL-15 and differentiate into cytotoxic T cells.^29^ Interestingly, though, there are some patients whose HCV-specific T cells are only weakly primed during the acute infection phase.^75^ Hepatitis C-infected patients also have increased numbers of CD4^+^ T_reg_ cells, possibly as a result of the HCV core protein inducing a T_reg_-like phenotype expressing the T_reg_ transcription factor Foxp3, together with immunosuppressive IL-10.^76^

The mechanisms by which CD8^+^ T cells become exhausted and/or deleted during viral infection are not completely understood. However, there are several mechanisms in addition to CD4^+^ T-cell dysfunction that may result in CD8^+^ T-cell exhaustion. First, prolonged exposure to viral antigen increases TCR signalling. Hepatitis B and C viruses may also downregulate TCR signalling by reducing expression of the TCR-associated signalling molecule, CD3ζ, leading to reduced downstream signalling.^77, 78^ Expression of the co-stimulatory molecule CD28, is reduced in HBV- and HCV-infected patients, leading to reduced effectiveness of TCR signalling.^77, 78^ On the contrary, expression of co-inhibitory molecules, including PD-1 and Tim-3, are elevated on virus-specific CD8^+^ T cells in response to HBV and HCV infection.^74^ Treatment of HCV-specific cytotoxic T-cell clones with the Tim-3 ligand, galectin-9, induced apoptosis of the T cells.^79^ Tim-3 gene polymorphisms have been linked to enhanced HBV surface antigen seroclearance.^80^ Hepatocyte expression levels of PD-1’s ligand, PD-L1, are also significantly elevated in response to HBV and HCV infection and may also contribute to the reduced T-cell function. However, as hepatocyte-expressed PD-L1 levels are also increased in autoimmune disease,^81^ it has been speculated that this may be a result of elevated inflammation.^74^

Expression of galectin-9, the ligand for Tim-3, is upregulated on KCs and monocytes by HCV and induces expansion of CD4^+^ T_regs_ in a TGF-β-dependent manner.^79^ Hepatitis C virus also downregulates monocyte production of IL-12 as a result of increasing Tim-3 expression.^82^ As discussed previously, IL-12 promotes increased CD4^+^ T_h_1 cell numbers.

### Non-viral-induced chronic liver disease

The risk of liver cancer is also increased in non-viral pathologies induced by alcohol and non-alcoholic steatohepatitis. Common features of these pathologies are fat deposition, chronic inflammation and elevated TNF, together with persistent injury. Chronic exposure of T cells to TNF downregulates CD3ζ-chain protein expression and results in reduced cell surface TCR complex and associated T-cell hyporesponsiveness as seen in chronic inflammation.^83^

Liver cancer may also be promoted in these pathologies by the free fatty acid, linoleic acid. Linoleic acid induces selective global CD4^+^ T-cell apoptosis as a result of increased mitochondrial oxidative stress, and CD4^+^ T-cell depletion promotes liver cancer in inducible liver-specific MYC oncogenic transgenic mice fed a methionine and choline-deficient diet.^84^ CD4^+^ T cells may attenuate liver cancer development by promoting CD8^+^ cytotoxic T-cell activity against tumour antigens. Another possible mechanism is that CD4^+^ T cells are involved in the resolution of inflammation as TGF-β promotes T_h_17 T-cell trans-differentiation into T_reg_ cells during inflammation resolution.^85^

The contribution of immune checkpoint signalling to the development of alcohol- and non-alcohol-induced chronic liver disease is relatively unexplored to date. PD-1 protein expression is elevated on CD4^+^ T cells in alcohol-related cirrhosis and further elevated by chronic systemic endotoxin in acute alcoholic hepatitis patients in the first reported investigation of immune checkpoint molecules in non-viral liver disease.^86^ Tim-3 is also upregulated in acute alcoholic hepatitis but not alcohol-related cirrhosis. Ethanol and endotoxin also promote enhanced PD-1 expression on CD4^+^ and CD8^+^ T cells, and T_regs_ cells *in vitro*. However, the effect of elevated PD-1 and Tim-3 on T-cell exhaustion was not examined in the study. PD-1, though, promotes inflammation with increased T_h_17 cell and neutrophil numbers resulting in enhanced hepatic injury in the murine bile duct ligation model.^87^

### Hepatocellular carcinoma

Immune surveillance of HCC is subverted by the hepatic cytokine and immune environment as well as the tumour cells present. Levels of immunosuppressive cytokines IL-10 and TGF-β are upregulated in HCC patients at the same time as pro-inflammatory IFN-γ levels are downregulated.^88^ An increased percentage of circulating and tumour-infiltrating CD8^+^ T cells express PD-1 while tumour cells and tumour-associated monocytes and neutrophils express upregulated PD-L1 and CD47 levels in HCC patients.^89, 90, 91, 92^ The numbers of myeloid-derived suppressor cells are also elevated in HCC patients.^88, 93^ Furthermore, activation of the *c-Myc* oncogene switches on PD-L1 and CD47 expression in hepatocytes,^94^ raising the possibility that oncogenes may directly regulate PD-L1 levels to subvert surveillance. Nonetheless, the elevated PD-L1:PD-1 interactions reduce CD4^+^ and CD8^+^ T-cell numbers and function, which subsequently attenuates immune surveillance. A novel PD-1-expressing B-cell subset whose numbers correlate with disease stage as well as early recurrence has also recently been identified in HCC.^95^ The PD-1^hi^ B cells produce IL-10 in response to PD-1:PD-L1 interaction and so consequently reduce CD8^+^ cytotoxic T-cell function. Thus the combined actions of cytokines in the tumour environment significantly reduce immune surveillance, allowing HCC to avoid eradication.

## Immune checkpoint inhibitor therapy and HCC development

It is apparent that immune evasion contributes to both early and later stages of HCC development. Immune-based monoclonal antibody therapy has become an attractive alternative to pursue in light of the recent promising outcomes from trials that involve immune checkpoint inhibitors in solid tumour cancer therapy. However, the use of these therapies in treatment at various stages of HCC development is in its infancy. We will discuss how the monoclonal antibody therapy has been used at various stages of HCC development to reduce incidence, from both preclinical and clinical perspectives.

### Immunotherapy in viral-induced chronic liver disease

Immunotherapeutic monoclonal antibody treatment of chronic HBV and HCV infection to reverse T-cell exhaustion is relatively unexplored. PD-L1-blockade enhanced IFN-γ production by CD8^+^ T cells adoptively transferred to HBV transgenic mice and also restored IFN-γ production by CD8^+^ T cells and viral clearance in the HCV core murine model.^96^ *In vitro* blocking of PD-L1 on HBV patient T cells enhanced both CD4^+^ and CD8^+^ T-cell production of IFN-γ.^97^ Likewise, *in vitro* blockade of Tim-3 on T cells isolated from HBV and HCV patients restores virus-specific CD8^+^ T-cell cytolytic responses, including IFN-γ expression and T-cell proliferation.^98, 99^ Despite these promising *in vitro* and *in vivo* results, treating HCV in non-human primates by PD-1-blockade led to mixed results with only one out of three chimpanzees attaining HCV suppression.^100^ However, improved outcomes may be achieved by combining the therapy with other immune interventions.

### Immunotherapy in non-viral-induced chronic liver disease

Immune checkpoint inhibitors have not been directly used to modify chronic inflammation in non-viral chronic liver disease to date. Instead, they have been used to enhance antibacterial immunity in alcoholic hepatitis. *In vitro* blockade of PD-1 and Tim-3 enhanced IFN-γ and reduced IL-10 production by peripheral blood mononuclear cells and increased neutrophil phagocytic capacity and *Escherichia coli*-stimulated oxidative burst.^86^

### Immunotherapy in HCC

Sorafenib, a receptor tyrosine kinase small-molecule inhibitor, is currently the gold standard and the only systemic therapy approved for treatment of unresectable advanced HCC, prolonging survival from 4.2 to 6.5 months in the Asia-Pacific study and from 7.9 to 10.7 months in the SHARP study.^101^ The differences in outcomes from the Asia-Pacific and SHARP studies may relate to the disease initiator, with HBV-related HCC accounting for 73% of patients in the Asia-Pacific study compared with 15.5% in the SHARP study. Patients with HCV-related HCC had the greatest benefit with sorafenib treatment prolonging survival from 7.4 to 14.0 months, suggesting its efficacy may depend on the underlying HCC aetiology.^101^ The modest survival benefit afforded by sorafenib may be partially derived via beneficial immune modulation, as discussed below, suggesting that combining sorafenib with other immune therapies may be beneficial in the treatment of HCC.

#### Sorafenib regulates hepatic immunity

Specifically, sorafenib blocks signalling via vascular endothelial growth factor receptor, platelet-derived growth factor receptor and C-Raf.^102, 103, 104^ Less well known is the ability of sorafenib to block colony-stimulating factor-1 receptor activity, which is required for survival, proliferation and differentiation of monocytes.^105^ A number of *in vitro* and *in vivo* studies have now reported that sorafenib also stimulates the immune system. Sorafenib directly upregulates IL-2 secretion by peripheral blood CD4^+^ T_regs_ isolated from HCC patients.^106^ Sorafenib also inhibited the suppressive properties and proliferation of CD4^+^ T_regs_ as well as enhanced their apoptosis in a preclinical orthotopic HCC model.^107^ This was not due to the reduction in tumour burden, as sorafenib also reduced CD4^+^ T_regs_ numbers in tumour-free mice. Further, sorafenib moderated the number of PD-1-expressing CD8^+^ T cells. Finally, sorafenib inhibited the activity of the disintegrin and metalloprotease member ADAM-9 to reduce MHC-1-related chain A shedding from tumour cells and enhanced NK cell activity.^108^ Hence, it is apparent that sorafenib may increase overall survival via immune effects as well as by the more widely known effects of reducing angiogenesis, arresting cell cycle and inducing apoptosis.

#### Immune checkpoint inhibitors

A recent study showed that anti-PD-1 therapy was more effective in non-small-cell lung cancer that had a higher nonsynonymous somatic tumour mutation burden.^109^ In general, tumour mutation rates are higher when an environmental mutagen is involved such as ultraviolet radiation in melanoma or cigarette smoke in lung cancer. Early studies in HCC suggest that tumour mutation burden is higher in melanoma and lung cancer but may depend upon the aetiology.^110, 111, 112^ Importantly and similar to melanoma and lung cancer, HCC does not appear to have an ‘oncogene addiction’.^113^ Thus it is reasonable to suggest immune checkpoint inhibitors as promising HCC treatments. Below, we examine the preclinical and clinical studies that have been reported thus far.

As discussed previously, expression of PD-1 and CD47 are upregulated in HCC. Anti-murine PD-1 antibody monotherapy reduced tumour size of the HCA1 orthotopic tumour model by approximately 50% in preclinical studies. However, the same anti-PD-1 antibody reduced tumour size by only 20% in mammalian sterile 20-like 1 (Mst1^−/−^Mst2^F/−^) mice in which hepatocarcinogenesis was induced by carbon tetrachloride. Eighteen out of the 41 advanced HCC patients with Child-Pugh score <B7 completed a 2-year Phase-I/II clinical safety trial of the PD-1 inhibitor, nivolumab (CheckMate 040; ClinicalTrials.gov number NCT01658878), although the dose cycle was not reported.^114^ Two patients had a complete response, while treatment of 23 patients was discontinued as a result of disease progression. Only two patients were discontinued owing to drug-related adverse events. This trial was extended to include a dose expansion arm of 3 mg kg^−1^ every 2 weeks in 214 patients.^115^ Complete response was achieved in three patients while partial response was achieved in 39 patients and disease was stable in a further 96 patients.^115^

The expression of CTLA-4 has not been examined in HCC and no preclinical studies involving CTLA4 blockade in HCC have been reported. Notwithstanding, a Phase II clinical trial blocking CTLA4 with a monoclonal antibody has been carried out in 21 patients with unresectable HCC of Child-Pugh class A or B (Clinicaltrials.gov number NCT01008358).^116^ Each patient received at least two treatment cycles of 90 days. Seventeen patients were evaluated for tumour response, with no complete responses and three patients with partial response that lasted for up to 15.8 months. Disease was stabilised in a further 10 patients with half stabilised for >6 months.^116^

CD47 is an innate immune checkpoint that binds to signal regulatory protein-α on monocytes, macrophages and DCs. CD47 inhibits phagocytic activity of these APCs by acting as a ‘don’t eat me’ signal. Antibody neutralisation of CD47 enhances phagocytosis of tumour cells and increases the durability of T-cell immune inhibitory therapy. Preclinical studies in various HCC xenograft models have mostly shown that monoclonal antibody inhibition of CD47 suppresses tumour growth.^117^ Further, CD47 blockade sensitises Huh7/MHCC-97L tumour cells to the chemotherapeutic drugs doxorubicin and cisplatin by increasing macrophage phagocytosis in *in vivo* murine zenografts.^118^ However, to the best of our knowledge, no clinical trials targeting CD47 in HCC have been initiated.

Second-generation immunotherapies against negative regulators of T-cell activation, including Tim-3 and Lag-3, are in the relatively early stages of clinical development for advanced solid tumours. Patients are currently being recruited for Phase I/IIa dose escalation and cohort expansion trials for Tim-3 and Lag-3 (ClinicalTrials.gov NCT02817633 and NCT01968109, respectively). The roles of Tim-3 and Lag-3 are relatively unexplored in HCC development. Tim-3 is expressed on an increased percentage of CD4^+^ and CD8^+^ T cells in HBV-induced HCC compared with the surrounding tissue and predicted a poor prognosis.^119^ Further, Tim-3 expression on tumour-associated macrophages in HBV-induced HCC stimulated tumour-promoting IL-6 production.^120^ Similarly, Lag-3 is also expressed on an increased percentage of CD8^+^ T cells in HBV-induced HCC and correlates with impaired HBV-specific CD8^+^ cytotoxicity in HCC patients.^121^ However, it remains to be seen how successful these second-generation immunotherapies will be in general.

Immune checkpoint molecules have critical roles in fine-tuning the immune system to maintain self-tolerance. As such, patients may develop immune-related adverse events ranging from common but mild symptoms such as fatique, nausea and skin rash to the severe but much less common such as hypothyroidism and the neurological conditions, Guillain–Barre syndrome and myasthenia gravis.^122^ Skin rash (65%), fatigue (55%) and diarrhoea (30%) were the most commonly reported immune-related adverse events in HCC patients receiving anti-CTLA-4 treatment.^116^ Hypothyroidism was only observed in 1 out of the 20 patients who underwent the anti-CTLA-4 treatment.^116^ Anti-PD-1 treatment of HCC patients resulted in less immune-related adverse events, with skin rash (23%), pruritis (19%) and diarrhoea (10%) most commonly reported.^115^

Another possible immune-related side effect of immune checkpoint inhibitor reactivation of T-cell cytotoxicity is an increase in hepatic damage. Anti-PD-1 (10 mg kg^−1^) treatment in a proof-of-concept study (NCT00703469) in 10 patients resulted in one Grade 4 elevation (>8 upper limits of normal) of serum alanine transaminase, although other indicators of hepatic function such as bilirubin and the international normalised ratio (that is, blood-clotting test) did not change. The elevated serum alanine transaminase coincided with the onset of a four-log reduction in HCV load and resolved without intervention.^123^ Anti-CTLA-4 treatment resulted in a Grade 3 elevation of serum transaminases after the first dose in more than half of the HCV-induced HCC patients but was not associated with a decline in liver function.^116^ Moreover, anti-CTLA-4 treatment also reduced the HCV load and tended to increase the numbers of IFN-γ-producing lymphocytes in the study by Sangro *et al.*^116^ Regardless, blockade of both CTLA-4 and PD-1 were concluded to be safe in treatment of HCC regardless of viral status.^115, 116^

#### Sorafenib and immune checkpoint inhibitors

As development of resistance is often the long-term response to sorafenib treatment, combination therapy with immune checkpoint inhibitors has been explored in preclinical studies. Sorafenib-resistant tumour cells derived from patient xenografts had elevated CD47 levels and CD47 blockade reduced their proliferation.^118^ PD-1 blockade, though, did not enhance sorafenib-reduced tumour growth in the orthotopic HCC implant and Mst-mutant mouse HCC models. However, PD-1 blockade together with inhibition of stromal cell-derived 1α receptor, C-X-C motif chemokine receptor 4, did significantly further reduce tumour growth in these two models.^124^ These preclinical data suggest that immune checkpoint inhibitor therapy combined with sorafenib may lead to improved outcomes.^115^ We await the outcome of the clinical trial combining sorafenib treatment with the anti-PD-1 monoclonal therapy, PDR001 (ClinicalTrials.gov number NCT02988440). Clinical trials of other currently approved therapies combined with immune checkpoint inhibitors are listed in Table 1.

## Conclusion

The role of the immune system in liver pathologies leading to HCC is complex and significant. The failure of many new drugs targeting HCC in phase III trials mandates that new and different approaches are more thoroughly explored. Immune checkpoint inhibitor therapy to stimulate the immune system has been demonstrated to be safe in HCC patients in the limited clinical data available. While showing promise, efficacy of the checkpoint inhibitors has been somewhat limited. The majority of preclinical studies of immune checkpoint inhibitors in HCC to date have used orthotopic or xenograft models to validate their efficacy. There is currently a profound lack of understanding of how the immune system controls the various stages of HCC development. Comprehensive expression profiling of checkpoint inhibitors together with detailed studies of how the immune system dampens HCC development during disease progression in models of different aetiologies that naturally develop HCC, while mimicking human disease need to be undertaken to realise the clinical potential of immune checkpoint inhibitor therapy. Importantly, these studies would enable the determination of clinical aetiologies and/or stage(s) for which the intervention will be most effective.

## Figures and Tables

**Table 1:** Clinical trials involving immune checkpoint inhibitors

*Target*	*Antibody*	*Phase*	*Trial ID*	*Sponsor*	*Combination treatment*	*Study population*	*Status*	*Results*
CTLA-4	CP-675,206	2	NCT01008358	Clinica Universidad de Navarra	NA	Patients with HCV-induced HCC not amenable to other therapies	Completed	^115^
	Tremelimumab	1	NCT01853618	National Cancer Institute	TACE, radiofrequency ablation, SBRT or chemoembolisation	Patients with advanced liver cancer	Ongoing/not recruiting	NA
	Tremelimumab	1/2	NCT02821754	National Cancer Institute	Durvalumab and TACE, radiofrequency ablation, SBRT or chemoembolisation	Patients with advanced liver cancer	Recruiting	NA
PD-1	Nivolumab	1/2	NCT01658878	Bristol-Myers Squibb & Ono Pharmaceutical Co. Ltd.	Part 1—safety; Part 2—comparison with sorafenib; Part 3—combination with Ipillimumab (CTLA-4)	Parts 1, 2—patients with uninfected HCC, HCV-infected HCC patients, HBV-infected HCC patients; Part 3—patients with advanced HCC	Recruiting	^114^
	Nivolumab	3	NCT02576509	Bristol-Myers Squibb & Ono Pharmaceutical Co. Ltd.	Comparison with sorafenib	Patients with advanced HCC	Recruiting	NA
	Nivolumab	1	NCT02837029	Northwestern University, Bristol-Myers Squibb and National Cancer Institute	Y90 glass microspheres	Patients with stages IIIA, IIIB, IIIC, IVA and IVB HCC	Recruiting	NA
	Nivolumab	1/2	NCT02859324	Celgene	CC-122 (pleitropic pathway modifier)	Patients with unresectable HCC	Recruiting	NA
	Nivolumab	2	NCT03033446	National Cancer Centre, Singapore	Y90 radioembolisation	Asian patients with advanced HCC	Recruiting	NA
	Nivolumab	1/2a	NCT03071094	Transgene	Pexa-Vec (JX-594 oncolytic virus)	Patients with advanced liver cancer	Not yet recruiting	NA
	Nivolumab	1	NCT03143270	Memorial Sloane Kettering Cancer Center	Drug eluting bead transarterial chemoembolisation	Patients with advanced HCC	Recruiting	NA
	Pembrolizumab	2	NCT02702414	Merck Sharp & Dohme Corp.	NA	Patients with previously systemically treated HCC	Ongoing/not recruiting	NA
	PDR001	1/2	NCT02795429	Novartis Pharmaceuticals	INC280 (c-Met)	Adult patients with advanced HCC	Recruiting	NA
	PDR001	1b	NCT02988440	Novartis Pharmaceuticals	Sorafenib	Adult patients with advanced HCC	Recruiting	NA
	SHR-1210	1/2	NCT02942329	The Affiliated Hospital of the Chinese Academy of Military Medical Sciences	Apatinib (VEGFRII)	Patients with HCC or gastric cancer	Recruiting	NA
	SHR-1210	2/3	NCT02989922	Jiangsu HengRui Medicine Co., Ltd	NA	Patients with non-resectable HCC who failed or did not tolerate prior systemic treatment	Recruiting	NA
	Pembrolizumab	1	NCT03099564	Hoosier Cancer Research Network	Y90 radioembolisation	Patients with poor prognosis HCC who are ineligible for liver transplant or surgical resection with well-compensated liver function	Recruiting	NA

Abbreviations: CTLA-4, cytotoxic T lymphocyte antigen 4; HBV, hepatitis B virus; HCC, hepatocellular carcinoma; HCV, hepatitis C virus; NA, not applicable; PD-1, programmed cell death protein-1; SBRT, stereotactic body radiation therapy; TACE, transarterial chemoembolization; VEGFR, vascular endothelial growth factor receptor.

## References

[bib1] Laursen L. A preventable cancer. Nature 2014; 516: S2–S3. 25470197 10.1038/516S2a

[bib2] Sangiovanni A, Colombo M. Treatment of hepatocellular carcinoma: beyond international guidelines. Liver Int 2016; 36 (Suppl 1): 124–129. 26725909 10.1111/liv.13028

[bib3] Maximin S, Ganeshan DM, Shanbhogue AK, Dighe MK, Yeh MM, Kolokythas O et al. Current update on combined hepatocellular-cholangiocarcinoma. Eur J Radiol Open 2014; 1: 40–48. 26937426 10.1016/j.ejro.2014.07.001 PMC4750566

[bib4] Kohn-Gaone J, Gogoi-Tiwari J, Ramm GA, Olynyk JK, Tirnitz-Parker JE. The role of liver progenitor cells during liver regeneration, fibrogenesis, and carcinogenesis. Am J Physiol 2016; 310: G143–154. 10.1152/ajpgi.00215.2015 26608186

[bib5] Rizvi S, Gores GJ. Pathogenesis, diagnosis, and management of cholangiocarcinoma. Gastroenterology 2013; 145: 1215–1229. 24140396 10.1053/j.gastro.2013.10.013 PMC3862291

[bib6] Chaudhary P, Bhadana U, Singh RA, Ahuja A. Primary hepatic angiosarcoma. Eur J Surg Oncol 2015; 41: 1137–1143. 26008857 10.1016/j.ejso.2015.04.022

[bib7] Lopez-Terrada D, Alaggio R, de Davila MT, Czauderna P, Hiyama E, Katzenstein H et al. Towards an international pediatric liver tumor consensus classification: Proceedings of the Los Angeles COG Liver Tumors Symposium. Mod Pathol 2014; 27: 472–491. 24008558 10.1038/modpathol.2013.80

[bib8] Hanahan D, Weinberg RA. Hallmarks of cancer: the next generation. Cell 2011; 144: 646–674. 21376230 10.1016/j.cell.2011.02.013

[bib9] Schreiber RD, Old LJ, Smyth MJ. Cancer immunoediting: integrating immunity's roles in cancer suppression and promotion. Science 2011; 331: 1565–1570. 21436444 10.1126/science.1203486

[bib10] Wilson CL, Jurk D, Fullard N, Banks P, Page A, Luli S et al. NFkappaB1 is a suppressor of neutrophil-driven hepatocellular carcinoma. Nat Commun 2015; 6: 6818. 25879839 10.1038/ncomms7818 PMC4410629

[bib11] Zabala M, Lasarte JJ, Perret C, Sola J, Berraondo P, Alfaro M et al. Induction of immunosuppressive molecules and regulatory T cells counteracts the antitumor effect of interleukin-12-based gene therapy in a transgenic mouse model of liver cancer. J Hepatol 2007; 47: 807–815. 17935823 10.1016/j.jhep.2007.07.025

[bib12] Tatsumi T, Takehara T, Kanto T, Miyagi T, Kuzushita N, Sugimoto Y et al. Administration of interleukin-12 enhances the therapeutic efficacy of dendritic cell-based tumor vaccines in mouse hepatocellular carcinoma. Cancer Res 2001; 61: 7563–7567. 11606395

[bib13] Vogt A, Sievers E, Lukacs-Kornek V, Decker G, Raskopf E, Meumann N et al. Improving immunotherapy of hepatocellular carcinoma (HCC) using dendritic cells (DC) engineered to express IL-12 *in vivo*. Liver Int 2014; 34: 447–461. 23998316 10.1111/liv.12284

[bib14] Heo J, Reid T, Ruo L, Breitbach CJ, Rose S, Bloomston M et al. Randomized dose-finding clinical trial of oncolytic immunotherapeutic vaccinia JX-594 in liver cancer. Nat Med 2013; 19: 329–336. 23396206 10.1038/nm.3089 PMC4268543

[bib15] Hoseini SS, Cheung NV. Immunotherapy of hepatocellular carcinoma using chimeric antigen receptors and bispecific antibodies. Cancer Lett 2017; 399: 44–52. 28428075 10.1016/j.canlet.2017.04.013 PMC5496242

[bib16] Redman JM, Gibney GT, Atkins MB. Advances in immunotherapy for melanoma. BMC Med 2016; 14: 20. 26850630 10.1186/s12916-016-0571-0 PMC4744430

[bib17] Yamane H, Paul WE. Early signaling events that underlie fate decisions of naive CD4^+^ T cells toward distinct T-helper cell subsets. Immunol Rev 2013; 252: 12–23. 23405892 10.1111/imr.12032 PMC3578301

[bib18] Kaplan MH, Hufford MM, Olson MR. The development and *in vivo* function of T helper 9 cells. Nat Rev Immunol 2015; 15: 295–307. 25848755 10.1038/nri3824 PMC4445728

[bib19] Qin SY, Lu DH, Guo XY, Luo W, Hu BL, Huang XL et al. A deleterious role for Th9/IL-9 in hepatic fibrogenesis. Sci Rep 2016; 6: 18694. 26728971 10.1038/srep18694 PMC4700496

[bib20] Aggarwal S, Gurney AL. IL-17: prototype member of an emerging cytokine family. J Leukoc Biol 2002; 71: 1–8. 11781375

[bib21] Hammerich L, Heymann F, Tacke F. Role of IL-17 and Th17 cells in liver diseases. Clin Dev Immunol 2011; 2011: 345803. 21197451 10.1155/2011/345803 PMC3010664

[bib22] Lafdil F, Miller AM, Ki SH, Gao B. Th17 cells and their associated cytokines in liver diseases. Cell Mol Immunol 2010; 7: 250–254. 20305686 10.1038/cmi.2010.5 PMC3732654

[bib23] Gomes AL, Teijeiro A, Buren S, Tummala KS, Yilmaz M, Waisman A et al. Metabolic inflammation-associated IL-17A causes non-alcoholic steatohepatitis and hepatocellular carcinoma. Cancer Cell 2016; 30: 161–175. 27411590 10.1016/j.ccell.2016.05.020

[bib24] Zou W. Regulatory T cells, tumour immunity and immunotherapy. Nat Rev Immunol 2006; 6: 295–307. 16557261 10.1038/nri1806

[bib25] Panduro M, Benoist C, Mathis D. Tissue Tregs. Ann Rev Immunol 2016; 34: 609–633. 27168246 10.1146/annurev-immunol-032712-095948 PMC4942112

[bib26] Shevach EM, Thornton AM. tTregs, pTregs, and iTregs: similarities and differences. Immunol Rev 2014; 259: 88–102. 24712461 10.1111/imr.12160 PMC3982187

[bib27] Mescher MF, Curtsinger JM, Agarwal P, Casey KA, Gerner M, Hammerbeck CD et al. Signals required for programming effector and memory development by CD8+ T cells. Immunol Rev 2006; 211: 81–92. 16824119 10.1111/j.0105-2896.2006.00382.x

[bib28] Boyman O, Purton JF, Surh CD, Sprent J. Cytokines and T-cell homeostasis. Curr Opin Immunol 2007; 19: 320–326. 17433869 10.1016/j.coi.2007.04.015

[bib29] Greyer M, Whitney PG, Stock AT, Davey GM, Tebartz C, Bachem A et al. T cell help amplifies innate signals in CD8^+^DCs for optimal CD8^+^ T cell priming. Cell Rep 2016; 14: 586–597. 26774484 10.1016/j.celrep.2015.12.058

[bib30] Laidlaw BJ, Craft JE, Kaech SM. The multifaceted role of CD4^+^ T cells in CD8^+^ T cell memory. Nat Rev Immunol 2016; 16: 102–111. 26781939 10.1038/nri.2015.10 PMC4860014

[bib31] Chen L, Flies DB. Molecular mechanisms of T cell co-stimulation and co-inhibition. Nat Rev Immunol 2013; 13: 227–242. 23470321 10.1038/nri3405 PMC3786574

[bib32] Keir ME, Butte MJ, Freeman GJ, Sharpe AH. PD-1 and its ligands in tolerance and immunity. Ann Rev Immunol 2008; 26: 677–704. 18173375 10.1146/annurev.immunol.26.021607.090331 PMC10637733

[bib33] Wherry EJ. T cell exhaustion. Nat Immunol 2011; 12: 492–499. 21739672 10.1038/ni.2035

[bib34] Freeman GJ, Long AJ, Iwai Y, Bourque K, Chernova T, Nishimura H et al. Engagement of the PD-1 immunoinhibitory receptor by a novel B7 family member leads to negative regulation of lymphocyte activation. J Exp Med 2000; 192: 1027–1034. 11015443 10.1084/jem.192.7.1027 PMC2193311

[bib35] Crawford A, Angelosanto JM, Kao C, Doering TA, Odorizzi PM, Barnett BE et al. Molecular and transcriptional basis of CD4^+^ T cell dysfunction during chronic infection. Immunity 2014; 40: 289–302. 24530057 10.1016/j.immuni.2014.01.005 PMC3990591

[bib36] Wherry EJ, Kurachi M. Molecular and cellular insights into T cell exhaustion. Nat Rev Immunol 2015; 15: 486–499. 26205583 10.1038/nri3862 PMC4889009

[bib37] Barber DL, Wherry EJ, Masopust D, Zhu B, Allison JP, Sharpe AH et al. Restoring function in exhausted CD8 T cells during chronic viral infection. Nature 2006; 439: 682–687. 16382236 10.1038/nature04444

[bib38] Jin HT, Anderson AC, Tan WG, West EE, Ha SJ, Araki K et al. Cooperation of Tim-3 and PD-1 in CD8 T-cell exhaustion during chronic viral infection. Proc Natl Acad Sci USA 2010; 107: 14733–14738. 20679213 10.1073/pnas.1009731107 PMC2930455

[bib39] Blackburn SD, Shin H, Haining WN, Zou T, Workman CJ, Polley A et al. Coregulation of CD8+ T cell exhaustion by multiple inhibitory receptors during chronic viral infection. Nat Immunol 2009; 10: 29–37. 19043418 10.1038/ni.1679 PMC2605166

[bib40] West EE, Jin HT, Rasheed AU, Penaloza-Macmaster P, Ha SJ, Tan WG et al. PD-L1 blockade synergizes with IL-2 therapy in reinvigorating exhausted T cells. J Clin Invest 2013; 123: 2604–2615. 23676462 10.1172/JCI67008 PMC3668811

[bib41] Brooks DG, Ha SJ, Elsaesser H, Sharpe AH, Freeman GJ, Oldstone MB. IL-10 and PD-L1 operate through distinct pathways to suppress T-cell activity during persistent viral infection. Proc Natl Acad Sci USA 2008; 105: 20428–20433. 19075244 10.1073/pnas.0811139106 PMC2629263

[bib42] Penaloza-MacMaster P, Kamphorst AO, Wieland A, Araki K, Iyer SS, West EE et al. Interplay between regulatory T cells and PD-1 in modulating T cell exhaustion and viral control during chronic LCMV infection. J Exp Med 2014; 211: 1905–1918. 25113973 10.1084/jem.20132577 PMC4144726

[bib43] Dietze KK, Zelinskyy G, Liu J, Kretzmer F, Schimmer S, Dittmer U. Combining regulatory T cell depletion and inhibitory receptor blockade improves reactivation of exhausted virus-specific CD8+ T cells and efficiently reduces chronic retroviral loads. PLoS Pathog 2013; 9: e1003798. 24339778 10.1371/journal.ppat.1003798 PMC3855586

[bib44] Wong YC, Tay SS, McCaughan GW, Bowen DG, Bertolino P. Immune outcomes in the liver: Is CD8 T cell fate determined by the environment? J Hepatol 2015; 63: 1005–1014. 26103545 10.1016/j.jhep.2015.05.033

[bib45] Tay SS, Wong YC, Roediger B, Sierro F, Lu B, McDonald DM et al. Intrahepatic activation of naive CD4+ T cells by liver-resident phagocytic cells. J Immunol 2014; 193: 2087–2095. 25070847 10.4049/jimmunol.1400037

[bib46] Bertolino P, Bowen DG, McCaughan GW, Fazekas de St Groth B. Antigen-specific primary activation of CD8+ T cells within the liver. J Immunol 2001; 166: 5430–5438. 11313380 10.4049/jimmunol.166.9.5430

[bib47] Grakoui A, Crispe IN. Presentation of hepatocellular antigens. Cell Mol Immunol 2016; 13: 293–300. 26924525 10.1038/cmi.2015.109 PMC4856799

[bib48] Scholzel K, Schildberg FA, Welz M, Borner C, Geiger S, Kurts C et al. Transfer of MHC-class-I molecules among liver sinusoidal cells facilitates hepatic immune surveillance. J Hepatol 2014; 61: 600–608. 24798625 10.1016/j.jhep.2014.04.028

[bib49] Thomson AW, Knolle PA. Antigen-presenting cell function in the tolerogenic liver environment. Nat Rev Immunol 2010; 10: 753–766. 20972472 10.1038/nri2858

[bib50] Knolle P, Schlaak J, Uhrig A, Kempf P, Meyer zum Buschenfelde KH, Gerken G. Human Kupffer cells secrete IL-10 in response to lipopolysaccharide (LPS) challenge. J Hepatol 1995; 22: 226–229. 7790711 10.1016/0168-8278(95)80433-1

[bib51] Horst AK, Neumann K, Diehl L, Tiegs G. Modulation of liver tolerance by conventional and nonconventional antigen-presenting cells and regulatory immune cells. Cell Mol Immunol 2016; 13: 277–292. 27041638 10.1038/cmi.2015.112 PMC4856800

[bib52] Goddard S, Youster J, Morgan E, Adams DH. Interleukin-10 secretion differentiates dendritic cells from human liver and skin. Am J Pathol 2004; 164: 511–519. 14742257 10.1016/S0002-9440(10)63141-0 PMC1602266

[bib53] Diehl L, Schurich A, Grochtmann R, Hegenbarth S, Chen L, Knolle PA. Tolerogenic maturation of liver sinusoidal endothelial cells promotes B7-homolog 1-dependent CD8^+^ T cell tolerance. Hepatology 2008; 47: 296–305. 17975811 10.1002/hep.21965

[bib54] Schildberg FA, Hegenbarth SI, Schumak B, Scholz K, Limmer A, Knolle PA. Liver sinusoidal endothelial cells veto CD8 T cell activation by antigen-presenting dendritic cells. Eur J Immunol 2008; 38: 957–967. 18383043 10.1002/eji.200738060

[bib55] Hammerich L, Tacke F. Interleukins in chronic liver disease: lessons learned from experimental mouse models. Clin Exp Gastroenterol 2014; 7: 297–306. 25214799 10.2147/CEG.S43737 PMC4158890

[bib56] Erhardt A, Biburger M, Papadopoulos T, Tiegs G. IL-10, regulatory T cells, and Kupffer cells mediate tolerance in concanavalin A-induced liver injury in mice. Hepatology 2007; 45: 475–485. 17256743 10.1002/hep.21498

[bib57] Taga K, Mostowski H, Tosato G. Human interleukin-10 can directly inhibit T-cell growth. Blood 1993; 81: 2964–2971. 8499633

[bib58] Knolle PA, Uhrig A, Hegenbarth S, Loser E, Schmitt E, Gerken G et al. IL-10 down-regulates T cell activation by antigen-presenting liver sinusoidal endothelial cells through decreased antigen uptake via the mannose receptor and lowered surface expression of accessory molecules. Clin Exp Immunol 1998; 114: 427–433. 9844054 10.1046/j.1365-2249.1998.00713.x PMC1905120

[bib59] Asadullah K, Sterry W, Volk HD. Interleukin-10 therapy—review of a new approach. Pharmacol Rev 2003; 55: 241–269. 12773629 10.1124/pr.55.2.4

[bib60] Schon HT, Weiskirchen R. Immunomodulatory effects of transforming growth factor-beta in the liver. Hepatobiliary Surg Nutr 2014; 3: 386–406. 25568862 10.3978/j.issn.2304-3881.2014.11.06 PMC4273109

[bib61] Gorelik L, Fields PE, Flavell RA. Cutting edge: TGF-beta inhibits Th type 2 development through inhibition of GATA-3 expression. J Immunol 2000; 165: 4773–4777. 11045997 10.4049/jimmunol.165.9.4773

[bib62] Gorelik L, Constant S, Flavell RA. Mechanism of transforming growth factor beta-induced inhibition of T helper type 1 differentiation. J Exp Med 2002; 195: 1499–1505. 12045248 10.1084/jem.20012076 PMC2193549

[bib63] Filippi CM, Juedes AE, Oldham JE, Ling E, Togher L, Peng Y et al. Transforming growth factor-beta suppresses the activation of CD8+ T-cells when naive but promotes their survival and function once antigen experienced: a two-faced impact on autoimmunity. Diabetes 2008; 57: 2684–2692. 18689691 10.2337/db08-0609 PMC2551678

[bib64] McKarns SC, Schwartz RH. Distinct effects of TGF-beta 1 on CD4+ and CD8+ T cell survival, division, and IL-2 production: a role for T cell intrinsic Smad3. J Immunol 2005; 174: 2071–2083. 15699137 10.4049/jimmunol.174.4.2071

[bib65] Thomas DA, Massague J. TGF-beta directly targets cytotoxic T cell functions during tumor evasion of immune surveillance. Cancer Cell 2005; 8: 369–380. 16286245 10.1016/j.ccr.2005.10.012

[bib66] Horras CJ, Lamb CL, Mitchell KA. Regulation of hepatocyte fate by interferon-gamma. Cytokine Growth Factor Rev 2011; 22: 35–43. 21334249 10.1016/j.cytogfr.2011.01.001 PMC3068863

[bib67] Kryczek I, Bruce AT, Gudjonsson JE, Johnston A, Aphale A, Vatan L et al. Induction of IL-17+ T cell trafficking and development by IFN-gamma: mechanism and pathological relevance in psoriasis. J Immunol 2008; 181: 4733–4741. 18802076 10.4049/jimmunol.181.7.4733 PMC2677162

[bib68] Lees JR. Interferon gamma in autoimmunity: a complicated player on a complex stage. Cytokine 2015; 74: 18–26. 25464925 10.1016/j.cyto.2014.10.014

[bib69] Luth S, Schrader J, Zander S, Carambia A, Buchkremer J, Huber S et al. Chronic inflammatory IFN-gamma signaling suppresses hepatocarcinogenesis in mice by sensitizing hepatocytes for apoptosis. Cancer Res 2011; 71: 3763–3771. 21512142 10.1158/0008-5472.CAN-10-3232

[bib70] Brenner D, Blaser H, Mak TW. Regulation of tumour necrosis factor signalling: live or let die. Nat Rev Immunol 2015; 15: 362–374. 26008591 10.1038/nri3834

[bib71] Bo X, Broome U, Remberger M, Sumitran-Holgersson S. Tumour necrosis factor alpha impairs function of liver derived T lymphocytes and natural killer cells in patients with primary sclerosing cholangitis. Gut 2001; 49: 131–141. 11413121 10.1136/gut.49.1.131 PMC1728361

[bib72] Beyer M, Abdullah Z, Chemnitz JM, Maisel D, Sander J, Lehmann C et al. Tumor-necrosis factor impairs CD4^+^ T cell-mediated immunological control in chronic viral infection. Nat Immunol 2016; 17: 593–603. 26950238 10.1038/ni.3399

[bib73] Fahey LM, Brooks DG. Opposing positive and negative regulation of T cell activity during viral persistence. Curr Opin Immunol 2010; 22: 348–354. 20381328 10.1016/j.coi.2010.03.004 PMC2891433

[bib74] Schmidt J, Blum HE, Thimme R. T-cell responses in hepatitis B and C virus infection: similarities and differences. Emerg Microbes Infect 2013; 2: e15. 26038456 10.1038/emi.2013.14 PMC3630955

[bib75] Rosen HR. Emerging concepts in immunity to hepatitis C virus infection. J Clin Invest 2013; 123: 4121–4130. 24084744 10.1172/JCI67714 PMC3784533

[bib76] Fernandez-Ponce C, Dominguez-Villar M, Aguado E, Garcia-Cozar F. CD4+ primary T cells expressing HCV-core protein upregulate Foxp3 and IL-10, suppressing CD4 and CD8 T cells. PLoS ONE 2014; 9: e85191. 24465502 10.1371/journal.pone.0085191 PMC3896374

[bib77] Maki A, Matsuda M, Asakawa M, Kono H, Fujii H, Matsumoto Y. Decreased expression of CD28 coincides with the down-modulation of CD3zeta and augmentation of caspase-3 activity in T cells from hepatocellular carcinoma-bearing patients and hepatitis C virus-infected patients. J Gastroenterol Hepatol 2004; 19: 1348–1356. 15610307 10.1111/j.1440-1746.2004.03455.x

[bib78] Das A, Hoare M, Davies N, Lopes AR, Dunn C, Kennedy PT et al. Functional skewing of the global CD8 T cell population in chronic hepatitis B virus infection. J Exp Med 2008; 205: 2111–2124. 18695005 10.1084/jem.20072076 PMC2526205

[bib79] Mengshol JA, Golden-Mason L, Arikawa T, Smith M, Niki T, McWilliams R et al. A crucial role for Kupffer cell-derived galectin-9 in regulation of T cell immunity in hepatitis C infection. PLoS ONE 2010; 5: e9504. 20209097 10.1371/journal.pone.0009504 PMC2831996

[bib80] Liao J, Zhang Q, Liao Y, Cai B, Chen J, Li L et al. Association of T-cell immunoglobulin and mucin domain-containing molecule 3 (Tim-3) polymorphisms with susceptibility and disease progression of HBV infection. PLoS ONE 2014; 9: e98280. 24867713 10.1371/journal.pone.0098280 PMC4035322

[bib81] Kassel R, Cruise MW, Iezzoni JC, Taylor NA, Pruett TL, Hahn YS. Chronically inflamed livers up-regulate expression of inhibitory B7 family members. Hepatology 2009; 50: 1625–1637. 19739236 10.1002/hep.23173 PMC2897253

[bib82] Zhang Y, Ma CJ, Wang JM, Ji XJ, Wu XY, Jia ZS et al. Tim-3 negatively regulates IL-12 expression by monocytes in HCV infection. PLoS ONE 2011; 6: e19664. 21637332 10.1371/journal.pone.0019664 PMC3102652

[bib83] Ersek B, Molnar V, Balogh A, Matko J, Cope AP, Buzas EI et al. CD3zeta-chain expression of human T lymphocytes is regulated by TNF via Src-like adaptor protein-dependent proteasomal degradation. J Immunol 2012; 189: 1602–1610. 22798681 10.4049/jimmunol.1102365

[bib84] Ma C, Kesarwala AH, Eggert T, Medina-Echeverz J, Kleiner DE, Jin P et al. NAFLD causes selective CD4^+^ T lymphocyte loss and promotes hepatocarcinogenesis. Nature 2016; 531: 253–257. 26934227 10.1038/nature16969 PMC4786464

[bib85] Gagliani N, Amezcua Vesely MC, Iseppon A, Brockmann L, Xu H, Palm NW et al. Th17 cells transdifferentiate into regulatory T cells during resolution of inflammation. Nature 2015; 523: 221–225. 25924064 10.1038/nature14452 PMC4498984

[bib86] Markwick LJ, Riva A, Ryan JM, Cooksley H, Palma E, Tranah TH et al. Blockade of PD1 and TIM3 restores innate and adaptive immunity in patients with acute alcoholic hepatitis. Gastroenterology 2015; 148: 590–602. 25479137 10.1053/j.gastro.2014.11.041

[bib87] Licata LA, Nguyen CT, Burga RA, Falanga V, Espat NJ, Ayala A et al. Biliary obstruction results in PD-1-dependent liver T cell dysfunction and acute inflammation mediated by Th17 cells and neutrophils. J Leukoc Biol 2013; 94: 813–823. 23883516 10.1189/jlb.0313137 PMC3774842

[bib88] Kalathil S, Lugade AA, Miller A, Iyer R, Thanavala Y. Higher frequencies of GARP^+^CTLA-4^+^Foxp3^+^ T regulatory cells and myeloid-derived suppressor cells in hepatocellular carcinoma patients are associated with impaired T-cell functionality. Cancer Res 2013; 73: 2435–2444. 23423978 10.1158/0008-5472.CAN-12-3381 PMC3645275

[bib89] Shi F, Shi M, Zeng Z, Qi RZ, Liu ZW, Zhang JY et al. PD-1 and PD-L1 upregulation promotes CD8^+^ T-cell apoptosis and postoperative recurrence in hepatocellular carcinoma patients. Int J Cancer 2011; 128: 887–896. 20473887 10.1002/ijc.25397

[bib90] Kuang DM, Zhao Q, Peng C, Xu J, Zhang JP, Wu C et al. Activated monocytes in peritumoral stroma of hepatocellular carcinoma foster immune privilege and disease progression through PD-L1. J Exp Med 2009; 206: 1327–1337. 19451266 10.1084/jem.20082173 PMC2715058

[bib91] Zhao Q, Xiao X, Wu Y, Wei Y, Zhu LY, Zhou J et al. Interleukin-17-educated monocytes suppress cytotoxic T-cell function through B7-H1 in hepatocellular carcinoma patients. Eur J Immunol 2011; 41: 2314–2322. 21674477 10.1002/eji.201041282

[bib92] He G, Zhang H, Zhou J, Wang B, Chen Y, Kong Y et al. Peritumoural neutrophils negatively regulate adaptive immunity via the PD-L1/PD-1 signalling pathway in hepatocellular carcinoma. J Exp Clin Cancer Res 2015; 34: 141. 26581194 10.1186/s13046-015-0256-0 PMC4652417

[bib93] Hoechst B, Ormandy LA, Ballmaier M, Lehner F, Kruger C, Manns MP et al. A new population of myeloid-derived suppressor cells in hepatocellular carcinoma patients induces CD4^+^CD25^+^Foxp3^+^ T cells. Gastroenterology 2008; 135: 234–243. 18485901 10.1053/j.gastro.2008.03.020

[bib94] Casey SC, Tong L, Li Y, Do R, Walz S, Fitzgerald KN et al. MYC regulates the antitumor immune response through CD47 and PD-L1. Science 2016; 352: 227–231. 26966191 10.1126/science.aac9935 PMC4940030

[bib95] Xiao X, Lao XM, Chen MM, Liu RX, Wei Y, Ouyang FZ et al. PD-1hi identifies a novel regulatory B-cell population in human hepatoma that promotes disease progression. Cancer Discov 2016; 6: 546–559. 26928313 10.1158/2159-8290.CD-15-1408

[bib96] Lukens JR, Cruise MW, Lassen MG, Hahn YS. Blockade of PD-1/B7-H1 interaction restores effector CD8^+^ T cell responses in a hepatitis C virus core murine model. J Immunol 2008; 180: 4875–4884. 18354211 10.4049/jimmunol.180.7.4875 PMC2904552

[bib97] Fisicaro P, Valdatta C, Massari M, Loggi E, Biasini E, Sacchelli L et al. Antiviral intrahepatic T-cell responses can be restored by blocking programmed death-1 pathway in chronic hepatitis B. Gastroenterology 2010; 138: 682–693. 19800335 10.1053/j.gastro.2009.09.052

[bib98] Golden-Mason L, Palmer BE, Kassam N, Townshend-Bulson L, Livingston S, McMahon BJ et al. Negative immune regulator Tim-3 is overexpressed on T cells in hepatitis C virus infection and its blockade rescues dysfunctional CD4^+^ and CD8^+^ T cells. J Virol 2009; 83: 9122–9130. 19587053 10.1128/JVI.00639-09 PMC2738247

[bib99] Wu W, Shi Y, Li S, Zhang Y, Liu Y, Wu Y et al. Blockade of Tim-3 signaling restores the virus-specific CD8(+) T-cell response in patients with chronic hepatitis B. Eur J Immunol 2012; 42: 1180–1191. 22539292 10.1002/eji.201141852

[bib100] Fuller MJ, Callendret B, Zhu B, Freeman GJ, Hasselschwert DL, Satterfield W et al. Immunotherapy of chronic hepatitis C virus infection with antibodies against programmed cell death-1 (PD-1). Proc Natl Acad Sci USA 2013; 110: 15001–15006. 23980172 10.1073/pnas.1312772110 PMC3773803

[bib101] Bruix J, Raoul JL, Sherman M, Mazzaferro V, Bolondi L, Craxi A et al. Efficacy and safety of sorafenib in patients with advanced hepatocellular carcinoma: subanalyses of a phase III trial. J Hepatol 2012; 57: 821–829. 22727733 10.1016/j.jhep.2012.06.014 PMC12261288

[bib102] Lyons JF, Wilhelm S, Hibner B, Bollag G. Discovery of a novel Raf kinase inhibitor. Endocr Relat Cancer 2001; 8: 219–225. 11566613 10.1677/erc.0.0080219

[bib103] Wilhelm SM, Carter C, Tang L, Wilkie D, McNabola A, Rong H et al. BAY 43-9006 exhibits broad spectrum oral antitumor activity and targets the RAF/MEK/ERK pathway and receptor tyrosine kinases involved in tumor progression and angiogenesis. Cancer Res 2004; 64: 7099–7109. 15466206 10.1158/0008-5472.CAN-04-1443

[bib104] Plaza-Menacho I, Mologni L, Sala E, Gambacorti-Passerini C, Magee AI, Links TP et al. Sorafenib functions to potently suppress RET tyrosine kinase activity by direct enzymatic inhibition and promoting RET lysosomal degradation independent of proteasomal targeting. J Biol Chem 2007; 282: 29230–29240. 17664273 10.1074/jbc.M703461200

[bib105] Ullrich K, Wurster KD, Lamprecht B, Kochert K, Engert A, Dorken B et al. BAY 43-9006/Sorafenib blocks CSF1R activity and induces apoptosis in various classical Hodgkin lymphoma cell lines. Br J Haematol 2011; 155: 398–402. 21517818 10.1111/j.1365-2141.2011.08685.x

[bib106] Cabrera R, Ararat M, Xu Y, Brusko T, Wasserfall C, Atkinson MA et al. Immune modulation of effector CD4^+^ and regulatory T cell function by sorafenib in patients with hepatocellular carcinoma. Cancer Immunol Immunother 2013; 62: 737–746. 23223899 10.1007/s00262-012-1380-8 PMC3863727

[bib107] Chen ML, Yan BS, Lu WC, Chen MH, Yu SL, Yang PC et al. Sorafenib relieves cell-intrinsic and cell-extrinsic inhibitions of effector T cells in tumor microenvironment to augment antitumor immunity. Int J Cancer 2014; 134: 319–331. 23818246 10.1002/ijc.28362

[bib108] Kohga K, Takehara T, Tatsumi T, Ishida H, Miyagi T, Hosui A et al. Sorafenib inhibits the shedding of major histocompatibility complex class I-related chain A on hepatocellular carcinoma cells by down-regulating a disintegrin and metalloproteinase 9. Hepatology 2010; 51: 1264–1273. 20099300 10.1002/hep.23456

[bib109] Rizvi NA, Hellmann MD, Snyder A, Kvistborg P, Makarov V, Havel JJ et al. Cancer immunology. Mutational landscape determines sensitivity to PD-1 blockade in non-small cell lung cancer. Science 2015; 348: 124–128. 25765070 10.1126/science.aaa1348 PMC4993154

[bib110] Alexandrov LB, Nik-Zainal S, Wedge DC, Aparicio SA, Behjati S, Biankin AV et al. Signatures of mutational processes in human cancer. Nature 2013; 500: 415–421. 23945592 10.1038/nature12477 PMC3776390

[bib111] Yao S, Johnson C, Hu Q, Yan L, Liu B, Ambrosone CB et al. Differences in somatic mutation landscape of hepatocellular carcinoma in Asian American and European American populations. Oncotarget 2016; 7: 40491–40499. 27246981 10.18632/oncotarget.9636 PMC5130022

[bib112] Fujimoto A, Totoki Y, Abe T, Boroevich KA, Hosoda F, Nguyen HH et al. Whole-genome sequencing of liver cancers identifies etiological influences on mutation patterns and recurrent mutations in chromatin regulators. Nat Genet 2012; 44: 760–764. 22634756 10.1038/ng.2291

[bib113] Llovet JM, Villanueva A, Lachenmayer A, Finn RS. Advances in targeted therapies for hepatocellular carcinoma in the genomic era. Nat Rev Clin Oncol 2015; 12: 408–424. 26054909 10.1038/nrclinonc.2015.103

[bib114] El-Khoueiry AB, Melero I, Crocenzi TS, Welling TH, Yau TC, Yeo W et al. Phase I/II safety and antitumor activity of nivolumab in patients with advanced hepatocellular carcinoma (HCC): CA209-040. ASCO Meeting Abstr 2015; 33: LBA101.

[bib115] El-Khoueiry AB, Sangro B, Yau T, Crocenzi TS, Kudo M, Hsu C et al. Nivolumab in patients with advanced hepatocellular carcinoma (CheckMate 040): an open-label, non-comparative, phase 1/2 dose escalation and expansion trial. Lancet 2017; 389: 2492–2502. 28434648 10.1016/S0140-6736(17)31046-2 PMC7539326

[bib116] Sangro B, Gomez-Martin C, de la Mata M, Inarrairaegui M, Garralda E, Barrera P et al. A clinical trial of CTLA-4 blockade with tremelimumab in patients with hepatocellular carcinoma and chronic hepatitis C. J Hepatol 2013; 59: 81–88. 23466307 10.1016/j.jhep.2013.02.022

[bib117] Xiao Z, Chung H, Banan B, Manning PT, Ott KC, Lin S et al. Antibody mediated therapy targeting CD47 inhibits tumor progression of hepatocellular carcinoma. Cancer Lett 2015; 360: 302–309. 25721088 10.1016/j.canlet.2015.02.036 PMC4886734

[bib118] Lo J, Lau EY, Ching RH, Cheng BY, Ma MK, Ng IO et al. Nuclear factor kappa B-mediated CD47 up-regulation promotes sorafenib resistance and its blockade synergizes the effect of sorafenib in hepatocellular carcinoma in mice. Hepatology 2015; 62: 534–545. 25902734 10.1002/hep.27859

[bib119] Li H, Wu K, Tao K, Chen L, Zheng Q, Lu X et al. Tim-3/galectin-9 signaling pathway mediates T-cell dysfunction and predicts poor prognosis in patients with hepatitis B virus-associated hepatocellular carcinoma. Hepatology 2012; 56: 1342–1351. 22505239 10.1002/hep.25777

[bib120] Yan W, Liu X, Ma H, Zhang H, Song X, Gao L et al. Tim-3 fosters HCC development by enhancing TGF-beta-mediated alternative activation of macrophages. Gut 2015; 64: 1593–1604. 25608525 10.1136/gutjnl-2014-307671

[bib121] Li FJ, Zhang Y, Jin GX, Yao L, Wu DQ. Expression of LAG-3 is coincident with the impaired effector function of HBV-specific CD8^+^ T cell in HCC patients. Immunol Lett 2013; 150: 116–122. 23261718 10.1016/j.imlet.2012.12.004

[bib122] Day D, Hansen AR. Immune-related adverse events associated with immune checkpoint inhibitors. BioDrugs 2016; 30: 571–584. 27848165 10.1007/s40259-016-0204-3

[bib123] Gardiner D, Lalezari J, Lawitz E, DiMicco M, Ghalib R, Reddy KR et al. A randomized, double-blind, placebo-controlled assessment of BMS-936558, a fully human monoclonal antibody to programmed death-1 (PD-1), in patients with chronic hepatitis C virus infection. PLoS ONE 2013; 8: e63818. 23717490 10.1371/journal.pone.0063818 PMC3661719

[bib124] Chen Y, Ramjiawan RR, Reiberger T, Ng MR, Hato T, Huang Y et al. CXCR4 inhibition in tumor microenvironment facilitates anti-programmed death receptor-1 immunotherapy in sorafenib-treated hepatocellular carcinoma in mice. Hepatology 2015; 61: 1591–1602. 25529917 10.1002/hep.27665 PMC4406806

